# Reproductive state-dependent cell turnover in the inner ear of the plainfin midshipman fish (*Porichthys notatus*)

**DOI:** 10.1242/jeb.250239

**Published:** 2025-03-19

**Authors:** Coty W. Jasper, Olivia Molano, Forrest Fearington, Joseph A. Sisneros, Allison B. Coffin

**Affiliations:** ^1^ Department of Integrative Physiology and Neuroscience, Washington State University Vancouver, Vancouver, WA 98686, USA; ^2^ Neuroscience Program, College of Arts and Sciences, Washington State University Vancouver, Vancouver, WA 98686, USA; ^3^ Department of Psychology, the Department of Biology and the Virginia Merrill Bloedel Hearing Research Center, University of Washington, Seattle, WA 98195-7923, USA

**Keywords:** Hair cell, Hearing, Auditory plasticity, Fish ear, Midshipman fish, Acoustic communication

## Abstract

Plainfin midshipman fish (*Porichthys notatus*) exhibit seasonal auditory plasticity that enhances their reproductive success. During the summer, type I male midshipman acoustically court females and both the males and females exhibit increased auditory sensitivity during this period. The enhanced auditory sensitivity is associated with increased density of sensory hair cells in the saccule but not the utricle, suggesting that different mechanisms underlie physiological plasticity in distinct inner ear regions. To better understand how shifts in hair cell number occur within auditory tissues, we examined cell turnover across breeding states and sexes in midshipman fish. We found that reproductive type I males exhibited less saccular cell proliferation than non-reproductive males without a change in cell death, indicating a net loss of saccular cells during the breeding season. By contrast, saccular cell proliferation increased in summer females, with no seasonal changes in other inner ear epithelia. Collectively, our data reveal that multiple mechanisms are likely to contribute to seasonal auditory plasticity within a single species, potentially within the ear of an individual animal.

## Teaser

**Summary:** Quantification of cell division and cell death in the inner ears of type I male and female midshipman fish reveals cellular plasticity in the midshipman inner ear.

## INTRODUCTION

Acoustic communication is vital for courtship and reproductive success across many vertebrates, with many species exhibiting reproductively dependent auditory plasticity that enhances the encoding of courtship calls (Caras et al., 2010; Gall et al., 2013; Goense and Feng, 2005; Miranda and Wilczynski, 2009; Sisneros, 2009; Sisneros and Bass, 2003). For instance, in some songbirds, auditory-evoked responses are amplified during the breeding season (Lucas et al., 2007). Similar reproductive state-dependent auditory plasticity is observed in the plainfin midshipman fish (*Porichthys notatus*) (Bhandiwad et al., 2017; Rogers et al., 2022; Rohmann and Bass, 2011; Sisneros, 2009; Sisneros and Bass, 2003).

Plainfin midshipman (midshipman) are a species of toadfish native to the Pacific coast of North America (Bass and Marchaterre, 1989). During the summer reproductive season, large nest-building type I males emit a prolonged ‘hum’ to attract females (Bass et al., 1996). Although the fundamental frequency of this call (90–100 Hz) does not propagate effectively in the shallow, rocky breeding environment, the higher harmonics contain significant acoustic energy, which females are likely to use to locate and select mates (Colleye et al., 2019; McKibben and Bass, 1998). Reproductive females are better at detecting these higher harmonics than their non-reproductive counterparts and the summer phenotype can be induced by steroid hormone treatment (Rogers et al., 2022; Sisneros, 2009; Sisneros and Bass, 2003; Sisneros et al., 2004a).

Similarly to most other fishes, the midshipman inner ear contains three end organs: the saccule, utricle and lagena, with the saccule considered the primary auditory organ in most species and the utricle traditionally associated with vestibular function (Fay and Popper, 1980; Sisneros, 2009; Tavolga et al., 1983). Saccular-evoked potential recordings from reproductive female midshipman show increased sensitivity (lower thresholds) relative to non-reproductive females, suggesting that seasonal auditory plasticity originates in the saccule (Sisneros, 2009). Interestingly, the utricle also serves an auditory function in midshipman and exhibits seasonal plasticity between female reproductive states, indicating that seasonal auditory plasticity occurs in multiple regions of the female midshipman inner ear (Rogers and Sisneros, 2020; Rogers et al., 2022). However, the mechanisms underlying this physiological plasticity are not fully understood.

Teleost fishes add new inner ear hair cells throughout their lifespan, with continuous turnover as older cells die and new cells are generated (Coffin et al., 2024; Lanford et al., 1996; Lombarte and Popper, 1994; Popper and Hoxter, 1984). In both female and type I male midshipman, decreased auditory thresholds during the breeding season are correlated with increased hair cells in the saccule, suggesting that reduced cell turnover likely underlies the morphological plasticity in this epithelium (Coffin et al., 2012; Lozier and Sisneros, 2019). Previous research showed consistent levels of cell death across all three inner ear end organs in female midshipman (Coffin et al., 2012). Based on these findings, we hypothesize that there is a significant increase in saccular cell proliferation associated with reproductive state.

In contrast, utricular hair cell density in both females and type I males is seasonally stable despite the physiological plasticity reported in females (Coffin et al., 2012; Rogers et al., 2022; comparable data for males are needed). We hypothesize that hair cell addition and death is seasonally stable in the utricle, suggesting that a mechanism independent of cell turnover is responsible for seasonal changes in utricular sensitivity.

## MATERIALS AND METHODS

### Fish collection

Reproductive state plainfin midshipman (*Porichthys notatus* Girard 1854) were collected from the Hood Canal in Washington State (USA) during the summer months (May to July) of 2020. During low tide, midshipman nests were located under rocks in the intertidal zone and the fish were collected directly from nests. Midshipman were then transported to Washington State University Vancouver (WSUV) within 3 h of collection and fish were processed for tissue collection within 24 h. Non-reproductive fish were collected in January 2023 via otter trawl in Monterey Bay, California (USA) and transported to WSUV. Winter fish were given a 2-week acclimation to recover from the stress of deep-water capture and transportation. Fish were housed in 32 ppm saltwater at a temperature of 14–16°C, with an 8 h:16 h light:dark cycle. Fish collection was conducted under the authorization of U.S. Fish and Wildlife, permit number SISNEROS 23-215. All procedures involving midshipman were approved by the Institutional Animal Care and Use Committee of Washington State University under protocol 6194.

We measured standard length (snout to base of caudal fin) in all animals. Standard lengths of the animals were as follows and are reported as mean±standard deviation (s.d.): non-reproductive females=17.04±1.90 cm; reproductive females=18.94±1.02 cm; non-reproductive males=9.91±0.65 cm; reproductive males=20.6 cm±4.51 cm. There was no difference in standard length between females from different seasons (unpaired *t*-test, *P*=0.06927). However, a significant difference in standard lengths was observed between non-reproductive and reproductive males (unpaired *t*-test, *P*=0.00013). Despite this difference, generalized linear models showed no significant effect of standard length on the number of positively labelled cells in male midshipman in either the cell proliferation (*P*=0.973) or cell death (*P*=0.738) assays, as described below.

We also analyzed gonadosomatic index (GSI), calculated as 100×gonad mass/(body mass−gonad mass) as a measure of relative reproductive state (Tomkins and Simmons, 2002). For reproductive females, mean GSI was 10.33±8.38 (±s.d.), while non-reproductive females had a mean GSI of 9.89±2.89. Although reproductive females exhibited a wide range of GSI values, we are confident that these fish were in reproductive condition, as they were collected directly from spawning nests during the summer. The presence of lower GSI in some summer females likely indicates that they had recently spawned. An unpaired two-sample *t*-test revealed no significant difference between the mean GSI values of females in different reproductive states (*P*=0.836). For reproductive males, the mean GSI was 1.07 (±0.68 s.d.), whereas non-reproductive males have regressed gonads with a mean GSI close to zero. Consequently, we did not conduct an analysis to determine if GSI was significantly different between the reproductive states of male midshipman.

### Tissue dissection

Following euthanasia using buffered Syncaine MS-222 (150–200 mg l^−1^, Pentair Aquatic Eco-systems), the heads of the midshipman were removed, and their inner ear cavities were opened to allow fluid access to the inner ear. The heads were then placed into 4% paraformaldehyde (Thermo Fisher Scientific) and stored overnight at 4°C to preserve the tissue. After fixation, the heads were rinsed three times in phosphate buffered saline (PBS) for 10 min each. The inner ears were then carefully dissected from midshipman heads, and the surrounding tissue was trimmed to expose the auditory epithelia for subsequent labeling, imaging and analysis.

### Cell proliferation analysis

To assess the rate of cell proliferation in midshipman ears across seasons, inner ear epithelia and sexes, we employed a 5-bromo-2-deoxyuridine (BrdU) incorporation assay. Fish were anesthetized with buffered MS-222 (15-20 mg l^−1^) and injected with 0.3 mg kg^−1^ BrdU (Sigma) in sterile PBS. Based on empirical observations, a 4 h incubation period following BrdU injection was sufficient to label proliferative cells in midshipman, similarly to findings in adult zebrafish (Coffin et al., 2024; Schuck and Smith, 2009). After BrdU incubation, fish were euthanized with MS-222 and decapitated. The heads were fixed and the tissue was dissected as previously described. To detect BrdU incorporation, ear tissues were first incubated in 1 mol l^−1^ HCl at 37°C for 1 h, then rinsed in 1 mol l^−1^ boric acid (pH 8.5) for 10 min, followed by three rinses in 0.5% Triton-X in PBS (PBST). The tissues were then immersed in a primary antibody solution containing of 0.5% PBST, 1% goat serum and 1% monoclonal mouse anti-BrdU primary antibody (MA3-071, Invitrogen). Tissue was incubated in this solution overnight at 4°C. Following primary antibody incubation, the tissues were then rinsed three times in 0.1% PBST and then placed in secondary antibody solution containing 0.1% PBST and 0.25% Alexa Fluor 488 nm goat anti-mouse secondary antibody (Invitrogen). The tissues were covered and placed on an orbital shaker for 4 h at room temperature. After secondary antibody application, the tissues were rinsed three times in PBS and mounted onto slides with Fluoromount G (Southern Biotech). Tissue was viewed using a Leica DRMB compound microscope with a 40× objective, resulting in a total magnification of 400×. We quantified the total number of BrdU-labelled cells in each epithelium using clearly identifiable epithelial boundaries (see Coffin et al., 2012, 2024). Therefore, counts represent all labelled cells for that end organ rather than discrete regions of interest.

### Cell death analysis

Cell death in the inner ear of type I males has not been previously studied. To quantify cell death across reproductive states in this sexual morph, we employed TUNEL labelling. Fish were euthanized and the tissue preserved as described above. Following inner ear dissection, a Sigma-Aldrich ApopTag Plus Fluorescein In Situ Apoptosis Detection Kit was used to visualize dying cells (Coffin et al., 2012). Fixed tissue was rinsed three times in PBS followed by incubation in 20 µg ml^−1^ proteinase K for 10 min at 37°C. Tissue was rinsed again in PBS, then incubated for 5 min in 33% acetic acid suspended in 100% ethanol, followed by additional PBS rinses. Rinsed tissues were placed in equilibration buffer for 30 s before being placed into reaction buffer with TdT enzyme for 1 h at 37°C in a humid environment. The enzymatic reaction was halted using stop-wash buffer for 10 min, followed by additional PBS rinses and a 1 h incubation at 37°C in blocking solution containing anti-digoxygenin. Finally, tissue was then rinsed in PBS and mounted onto slides using Fluoromount G. As with the BrdU assay, we quantified all TUNEL+ cells within each epithelium using a Leica DRMB compound microscope using 400× total magnification. For females, we utilized TUNEL data from Coffin et al. (2012).

### Statistical analysis

Statistical analyses were conducted using R version 4.2.0 (r-project.org). Cell proliferation and cell death data were examined using Shapiro and Levene tests followed by two-way ANOVA and Tukey-HSD *post hoc* analyses when appropriate. A Kruskal–Wallis test was used when the data were not normally distributed. Data are presented as mean±s.d. Each fish was treated as an experimental unit and only contributed one epithelium per analysis. Sample sizes (number of fish) are as follows: Female summer, *n*=13; female winter, *n*=7; male summer, *n*=9; male winter, *n*=12. For male animals, one ear per fish was used for BrdU labeling, the other for TUNEL labeling (TUNEL data for female animals was taken from Coffin et al., 2012). In some cases, there was dissection damage to an epithelium and we removed that sample from the final analysis. Therefore, sample sizes for a given group and assay are not identical for all epithelia. Sample sizes for each group and assay are given in the figure legends. Data are graphed to show each fish as an individual dot, where each data point (dot) represents the total number of labelled cells observed in an epithelium.

## RESULTS AND DISCUSSION

### Cell proliferation varies by season, sex and inner ear end organ

**Fig. 1.**
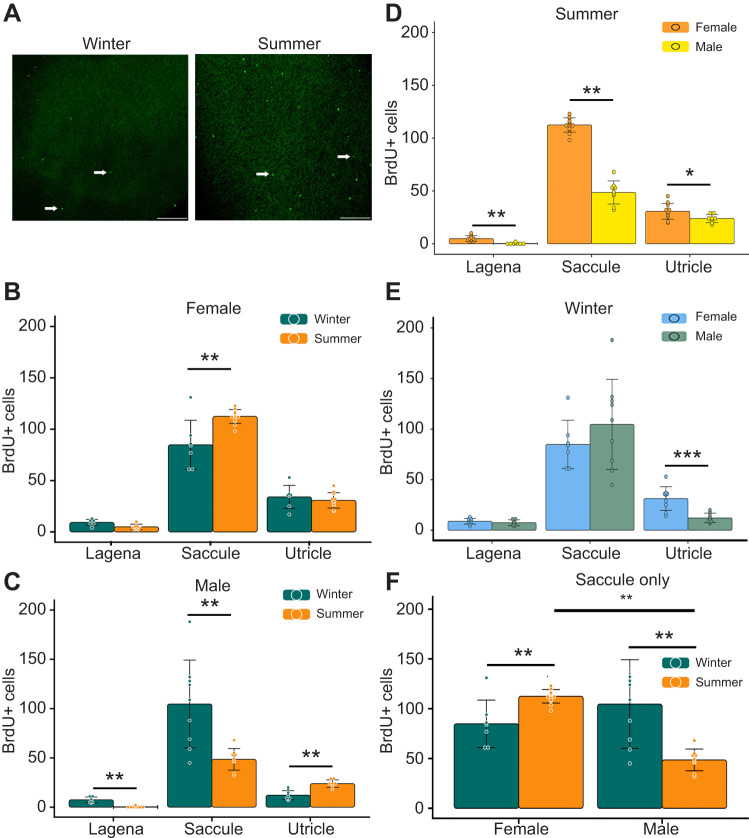
**Cell proliferation in the inner ear of the plainfin midshipman differs between season, epithelium and sex.** (A) Representative images of BrdU+ cells in the female saccule from winter (left) and summer (right). Arrows show BrdU+ cells; scale bars: 100 μm. (B,C) Quantification of BrdU+ cells by epithelium from (B) females and (C) males. (B) In females, there is a significant effect of season (*F*_1,58_=29.66, *P*<0.001), epithelium (*F*_2,57_=456.59, *P*<0.001) and their interaction (*F*_2,54_=13.68, *P*<0.0001) with more cell proliferation in summer saccules. Summer, *N*=11–13 fish; winter, *N*=7. (C) In males, there is a significant effect of season (*F*_1,45_=10.3, *P*=0.00246), epithelium (*F*_2,48_=68.31, *P*<0.001) and their interaction (*F*_2,45_=14.11, *P*<0.0001). Summer utricles had significantly more BrdU+ cells than winter while the saccule and lagena had fewer BrdU+ cells in the summer. *N*=8–9 fish per season. (D–F) Comparison between sexes. (D) In summer, there is a significant effect of sex (*F*_1, 58_=270.7, *P*<0.001), epithelium (*F*_2,57_=908.2, *P*<0.001) and their interaction (*F*_2,54_=183.3, *P*<0.001) with females having significantly higher cell proliferation than males. (E) In winter, there is no significant effect of sex (*F*_1,49_=0.57, *P*=0.4542). However, there is significant main effect of epithelium (*F*_2,48_=79.637, *P*<0.001), and the interaction term (*F*_2,45_=3.469, *P*=0.0397). (F) Sex and season significantly affect cell proliferation in the saccule (*F*_1,34_=25.995, *P*<0.0001). Females from either reproductive state had higher cell proliferation than summer males. All data are presented as means±s.d., dots represent individual epithelia. Data were analyzed by two-way ANOVA followed by Tukey-HSD (*P<0.05, **P<0.01, ***P<0.001).

Saccular hair cell density changes seasonally in female midshipman fish, with a significant increase during the summer and no corresponding change in cell death (Coffin et al., 2012). Our BrdU assay revealed a significant increase (24.6%) in cell proliferation in female saccules during the summer, but no significant changes in the utricle or lagena (Fig. 1A,B). This observation supports our hypothesis that increased cell proliferation may underlie increased saccular hair cell density.

Type I males also experience increased hair cell density during the summer but cell turnover has not been studied (Lozier and Sisneros, 2019). We found a significant 46.3% decrease in saccular cell proliferation during the summer compared with winter (Fig. 1C). This finding suggests that despite the higher saccular hair cell density in type I males in summer, cell proliferation declines. Similarly, type I male lagenae showed a modest 5% decrease in cell proliferation during the summer compared with winter (Fig. 1C). However, there was a notable 48% increase in BrdU+ utricular cells during the summer (Fig. 1C). Thus, utricles were the only epithelium to exhibit increased cell proliferation during the summer in type I males, while the lagena and saccule experienced a reduction. These findings do not support the hypothesis that saccular cell proliferation increases in reproductive-state type I males.

We then compared inner ear cell proliferation between reproductive states in females and type I males (Fig. 1D–F). Interestingly, saccules from reproductive type I males had 47.5% fewer dividing cells than non-reproductive females and 60.4% fewer BrdU+ cells than reproductive females. However, saccules from non-reproductive type I males did not significantly differ from those of females in either breeding condition. Additionally, reproductive females showed significantly more proliferation in both the lagena and utricle compared with reproductive type I males (Fig. 1D,E). In non-reproductive fish, a significant difference was observed only in the utricle, where females had more BrdU+ cells than males (Fig. 1D,E,F). Collectively, our data show that inner ear cell proliferation is generally higher in females than type I males, regardless of epithelium or reproductive state.

### Cell death is similar across sexes and seasons

**Fig. 2.**
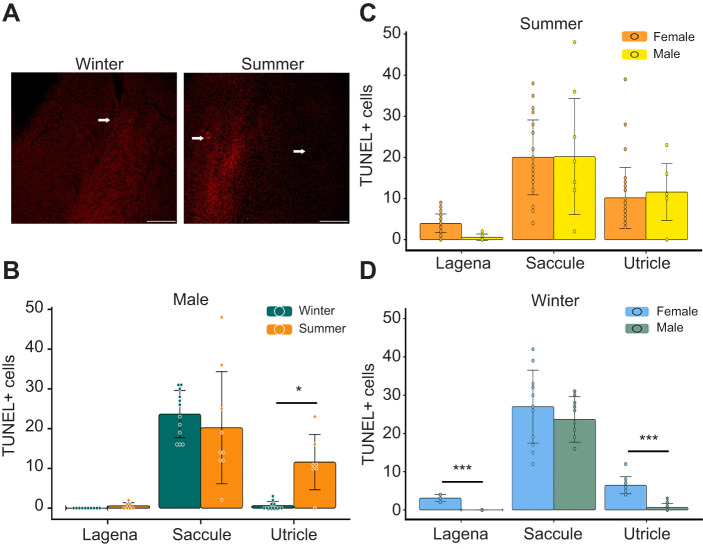
**Cell death in the inner ear of the plainfin midshipman varies between season, epithelium and sex.** (A) Representative images of TUNEL staining in the male saccule from winter (left) and summer (right). Arrows denote TUNEL+ cells; scale bars: 100 μm. (B) Quantification of TUNEL+ cells in males. Season had no significant effect on cell death (Kruskal–Wallis test, chi squared=1.556, *P*=0.2126). Summer, *N*=7–9 fish; winter, *N*=10–12. (C,D) TUNEL comparison between sexes. Female data from Coffin et al. (2012). (C) There is a significant effect of epithelium in summer fish (*F*_2,97_=37.674, *P*<0.001), but not sex (*F*_1,98_=0.069, *P*=0.794), nor their interaction (*F*_2,94_=0.584, *P*=0.560). (D) There is a significant effect of sex (*F*_1,65_=11.632, *P*<0.0012) and epithelium (*F*_2,64_=167.421, *P*<0.001) but not their interaction (*F*_2,61_=0.525, *P*=0.594), in winter animals. For all graphs, data are presented as means±s.d., dots represent individual epithelia (*P<0.05, ***P<0.001).

Using TUNEL labeling, we found no seasonal difference in saccular cell death in type I males (Fig. 2A,B; winter, 23.67±5.94; summer, 20.22±14.1; mean±s.d.), consistent with previous observations in females (Coffin et al., 2012). In type I males, lagenar hair cell death showed no substantial seasonal differences (winter, 0±0; summer, 0.57±0.79). Interestingly, a significant increase in utricular hair cell death was observed during the summer (winter, 0.64±1.0; summer, 11.97±6.94).

We compared our TUNEL results from type I males with previously published female data from Coffin et al. (2012) (Fig. 2C,D). While cell death was higher in female lagenae in the winter (compared with type I males) lagenar cell death remained low overall (ranges of 5–10 in females, 0–1 in males). Notably, we observed no sex differences in saccular cell death across seasons, which contrasts with our cell proliferation analysis. During the summer, there was no significant difference in utricular cell death between sexes. However, in non-reproductive fish, utricular cell death was significantly higher in females than in males (4–12 in females, 0–3 in males), which was similar to our cell proliferation analysis.

### Conclusions and implications

We identified seasonal and sex-dependent differences in cell proliferation and cell death within inner ears in midshipman fish. Our data suggest that hair cell addition in the saccule of female midshipman likely occurs through a proliferative mechanism, coincident with a period of enhanced sound encoding, suggesting that cell proliferation may drive hair cell addition and subsequent improvements in auditory sensitivity.

#### Seasonal cell turnover in female midshipman

The significant increase in cell proliferation in the saccule of reproductive females likely explains the findings of Coffin et al. (2012), who reported a significant increase in hair cell density within the summer female saccule without a corresponding change in cell death. Although Coffin et al. (2012) did not observe a significant seasonal change in cell proliferation within female saccules, they used the M-phase marker phosphorylated histone 3 (ph3), while we utilized the S-phase marker BrdU. S-phase of the cell cycle has a much longer duration than M-phase, increasing the probability of detecting cell division using an S-phase marker (Bernard et al., 2010). Coffin et al. (2012) also noted significantly more small, likely immature, hair bundles in several regions of the saccule in reproductive females. Therefore, female midshipman seasonally increase saccular hair cell numbers through a proliferative mechanism.

In contrast to the saccule, cell proliferation did not seasonally differ within the female utricle. Our data support the findings of Coffin et al. (2012), who reported no seasonal variation in utricular hair cell density. Interestingly, female midshipman collected during the summer exhibit increased utricular sensitivity between 65 and 400 Hz, despite stable hair cell numbers, suggesting that physiological plasticity is independent of hair cell addition in this epithelium (Rogers et al., 2022).

Several mechanisms could contribute to shifts in auditory thresholds. For example, the number of functional large conductance calcium-activated potassium (BK) channels within a hair cell positively correlates with frequency tuning and auditory sensitivity in several vertebrates, and saccular injection of a BK antagonist reduces saccular thresholds in non-reproductive female midshipman (Art et al., 1995; Fettiplace and Fuchs, 1999; Navaratnam et al., 1997; Pyott and Duncan, 2016; Rohmann et al., 2013; Smotherman and Narins, 1999). Additionally, dopamine signaling alters saccular thresholds in midshipman (Perelmuter et al., 2019). Therefore, reduced availability of functional BK channels or increased dopamine signaling in summer female saccules could revert threshold phenotypes to winter-like states.

However, multiple mechanisms may contribute to reduced auditory thresholds in reproductive females, including hair cell addition. There is a positive correlation between hair cell number and auditory sensitivity in the inner ears of many vertebrates, including fish (Coffin et al., 2024; Wang et al., 2015; Zeng et al., 2021). We hypothesize that in female midshipman, increased cell proliferation increases hair cell density during the breeding season, contributing to seasonal saccular threshold shifts. By contrast, utricular threshold shifts may be established through BK channel and/or dopamine-related mechanisms. Future studies are needed to understand the extent to which changes in hair cell density, BK function and dopamine signaling influence thresholds in the saccule versus the utricle.

#### Seasonal cell turnover in type I male midshipman

Type I males exhibit a seasonal decrease in saccular thresholds during the summer, coincident with increased saccular hair cell number (Lozier and Sisneros, 2019; Rohmann and Bass, 2011). We hypothesized that reproductive type I males would also show increased saccular cell proliferation, similarly to females. Contrary to our hypothesis, we observed that summer type I males had reduced cell proliferation and increased cell death within the saccule compared with winter. Additionally, saccular proliferation in winter type I males was not significantly different from females collected in either season. It is possible that size differences between type I and female epithelia contribute to the observed sex differences. However, we think it is unlikely that epithelium size would alter cell turnover, as previous studies have shown that hair cell density is not correlated with fish size in midshipman and the epithelium grows through the life of the fish (Coffin et al., 2012).

Type I males endure substantial environmental stress during the breeding season, which likely affects cell turnover. Type I males, but not females, remain in the nests throughout the breeding season to guard fertilized eggs (Brantley and Bass, 1994). During low tide, these males may be exposed to stagnant water, leading to high environmental temperatures (personal observation). Therefore, summer heat stress and/or reduced oxygen availability may contribute to the increased cell death observed in type I male utricles compared with summer females or winter males (Carmeliet et al., 1998; Payne et al., 2002).

Despite reduced cell proliferation, summer type I males exhibit increased hair cell density and enhanced auditory sensitivity, coinciding with a peak in circulating steroid hormones (Lozier and Sisneros, 2019; Rohmann and Bass, 2011; Sisneros et al., 2004b). We hypothesize that in type I males, cell proliferation and hair cell addition spikes during the pre-nesting season (April–May), then declines due to environmental stress throughout the breeding season (June–July). Owing to pandemic-related delays during the 2020 field season, our type I males were collected in July, likely after prolonged exposure to environmental stress. Future studies should investigate cell death and proliferation at multiple time points throughout the pre-nesting and nesting periods. Also, as our study used fish from one summer and one winter collecting season, it is possible that our findings are influenced by environmental conditions at the time of collection. Further study using fish collected over multiple seasons is therefore warranted.

Reproductive type I males had higher levels of utricular cell proliferation and death compared with levels in males in winter. Increased proliferation during the summer may maintain stable hair cell numbers despite increased cell death, implying that utricular function is important for reproductive success in type I males. Our findings align with the hypothesis proposed by Rogers and Sisneros (2020) that type I males may use utricles to detect grunts and hums of other males. These findings suggest that multiple mechanisms regulate cellular and physiological auditory plasticity, which may enhance acoustic communication and, consequently, reproductive success.
